# Photocatalytic Activities of Copper Doped Cadmium Sulfide Microspheres Prepared by a Facile Ultrasonic Spray-Pyrolysis Method

**DOI:** 10.3390/molecules21060735

**Published:** 2016-06-15

**Authors:** Jinzhan Su, Tao Zhang, Yufeng Li, Yubin Chen, Maochang Liu

**Affiliations:** International Research Center for Renewable Energy, State Key Laboratory of Multiphase Flow in Power Engineering, Xi’an Jiaotong University, No. 28, Xianning West Road, Xi’an 710049, Shaanxi, China; zt.268@stu.xjtu.edu.cn (T.Z.); lyf1127@stu.xjtu.edu.cn (Y.L.); ybchen@mail.xjtu.edu.cn (Y.C.); maochangliu@mail.xjtu.edu.cn (M.L.)

**Keywords:** CdS, doping, photocatalytic, spray pyrolysis

## Abstract

Ultrasonic spray pyrolysis is a superior method for preparing and synthesizing spherical particles of metal oxide or sulfide semiconductors. Cadmium sulfide (CdS) photocatalysts with different sizes and doped-CdS with different dopants and doping levels have been synthesized to study their properties of photocatalytic hydrogen production from water. The CdS photocatalysts were characterized with scanning electron microscopy (SEM), X-ray fluorescence-spectrometry (XRF), UV-Vis absorption spectra and X-ray diffraction (XRD) to study their morphological and optical properties. The sizes of the prepared CdS particles were found to be proportional to the concentration of the metal nitrates in the solution. The CdS photocatalyst with smaller size showed a better photocatalytic activity. In addition, Cu doped CdS were also deposited and their photocatalytic activities were also investigated. Decreased bandgaps of CdS synthesized with this method were found and could be due to high density surface defects originated from Cd vacancies. Incorporating the Cu elements increased the bandgap by taking the position of Cd vacancies and reducing the surface defect states. The optimal Cu-doped level was found to be 0.5 mol % toward hydrogen evolution from aqueous media in the presence of sacrificial electron donors (Na_2_S and Na_2_SO_3_) at a pH of 13.2. This study demonstrated that ultrasonic spray pyrolysis is a feasible approach for large-scale photocatalyst synthesis and corresponding doping modification.

## 1. Introduction

Photocatalytic water splitting has continuously been a hot academic topic since Fujishima and Honda′s work in hydrogen production from water using a TiO_2_ electrode [1]. Over the past decades, metal sulfides [2] and chalcogenides [3] have attracted broad interest from scientists engaged in photocatalysis. Of those compounds, cadmium sulfide (CdS) is an attractive semiconductor photocatalyst under continuous research due to its optimal band gap and suitable position of the conduction band and valence band edge [4,5,6]. Various CdS nanostructures or combinations were applied as photocatalysts for solar hydrogen production [7,8,9]. One drawback of this material is the photocorrosion by the photogenerated holes from the valence band of the CdS during the photocatalytic reaction in aqueous solution. In order to overcome the inherent disadvantages of CdS photocatalyst for hydrogen production, hybrid composites or solid solution based on CdS have been explored to improve the separation of photogenerated holes and electrons as well as their stabilities, and thus promote the overall photocatalytic activity [10,11,12,13,14,15]. Some photocatalysts with unique structure show promising efficiency and potential for large scale photocatalytic water splitting application [16]. Many novel methods have been applied for preparing and synthesizing CdS particles. Bao *et al.* [17] prepared nanoporous CdS nanostructrues by method of self-templanted synthesis, having a high hydrogen yield under visible light irradiation. Many other techniques such as hydrothermal method [18], thermal evaporation [19], biogenic synthesis [20], chemical bath deposition [21] were applied to prepare CdS nanostructures. It is reported that spray pyrolysis is an efficient way to prepare sulfides or oxides microspheric powder or films [22,23,24]. With this method, one can synthesize micro-particles of different sizes which range from submicrometers to micrometers by controlling the various concentrations of metal nitrates in their starting aqueous solution. Kikuo Okuyama *et al*. [22] prepared CdS fine particles with different particle sizes by an Ultrasonic spray-pyrolysis method. In Kikuo’s work, ZnS and CdS fine particles with different particle sizes have been prepared and the effects of temperature profile in the reactor furnace and concentration of the metal nitrates in the solution on the properties of prepared particles were also investigated.

Doping is a viable approach for modulating electrical, optical or photocatalytical properties of photocatalyst [25]. It can be used to reduce the band gap of photocatalyst by forming intermediate bands [26] or improve the chemical stability by injecting the holes into the formed acceptor energy level and reduce their activity on photocorrosion. [27] In our previous report, Cu doped CdS thin films were deposited by ultrasonic spray pyrolysis (USP). The non-uniform distribution of Cu atoms in the host CdS films formed disordered local p-n junctions which facilitate charge separation [28].

In addition, it has been reported that the USP method is a simple and cost-effective technique to prepare photocatalyst with different doping levels. A. Rmili *et al.* [29] prepared the undoped and Ni-doped CdS thin films with various doping levels by spray pyrolysis technique. The spray pyrolysis technique is considered as one of the most promising techniques for producing large scale inexpensive CdS catalyst for massive solar hydrogen application.

In this study, we synthesized the spherical CdS nanoparticles with different sizes profile and Cu doped CdS photocatalyst with different doping levels by ultrasonic spray pyrolysis. The dopants were added directly into the precursor for spray pyrolysis. The temperature in the tube-furnace was kept constant while the concentrations of metal nitrates, dopants and in the precursors containing Cd(NO_3_)_2_·4H_2_O and SC(NH_2_)_2_ were adjusted to change the particle size or doping level. The UV-Vis absorption spectrum, crystalline phase, fluorescence properties and photocatalystic activities of CdS or doped CdS photocatalysts were tested to investigate the effect of particle-sizes, dopant and doping level on their structural, optical and photocatalytic properties.

## 2. Results and Discussion

### 2.1. Morphological, Optical and Structural Properties of Undoped CdS Particles

**Figure 1:**
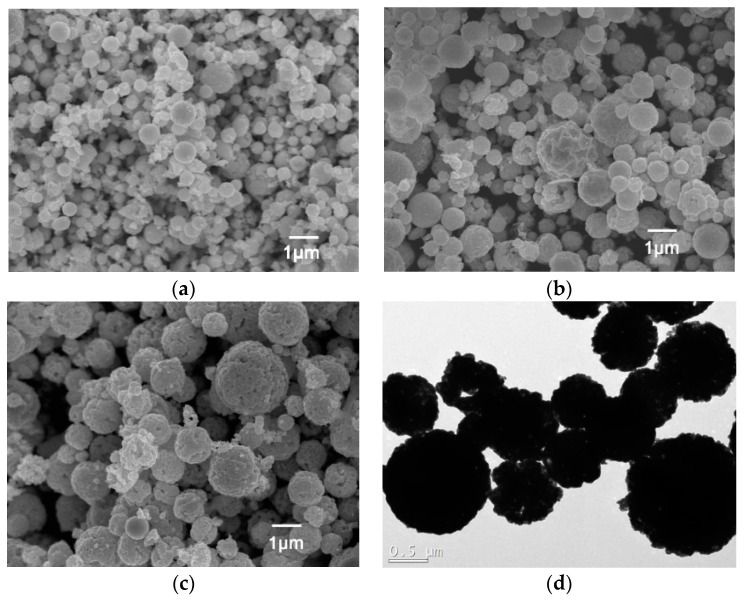
Scanning electron microscope images of CdS particles obtained from different starting-solution concentrations: (**a**) 0.1 mol/L; (**b**) 0.5 mol/L; (**c**) 1.0 mol/L and (**d**) TEM images of CdS particles with concentrations of 1.0 mol/L.

Figure 1 shows SEM images of spherical CdS particles obtained from different starting-solution concentrations at a constant pyrolysis temperature of 500 °C. It was found that the particle sizes increase with increasing starting-solution concentration and the surface of CdS particles is rather rough because it originates from aggregation of primary particles [22]. In addition, it is observed that the CdS particles prepared with same concentration possess different sizes. This can be due to the collision between precursor droplets and creating droplets with different sizes during the deposition. However, the average particle size can be controlled by changing the concentration of the starting solution. The TEM images shown in Figure 1d confirmed the rough surface of CdS particles and ununiformed sizes of obtained CdS particles.

**Figure 2:**
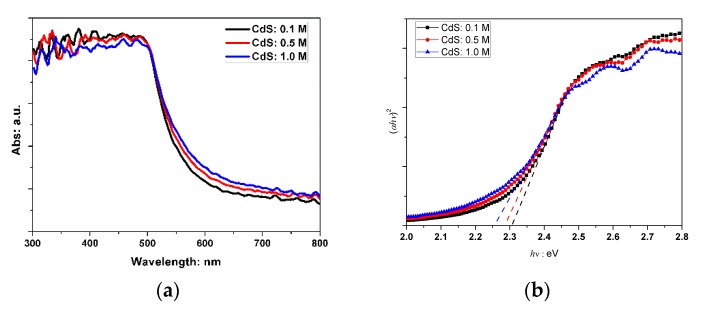
The UV-Vis absorption spectra (**a**) and corresponding Tauc plot (**b**) of CdS with different starting-solution concentrations.

Figure 2 shows the UV-Vis absorption spectra of prepared CdS nanoparticles with different starting-solution concentrations. The direct band gap (E_g_) can be determined using the Tauc relation given by [30]:

(αhν)^2^ = A × (hν − E_g_) (1)

where A is a constant, α is the absorption coefficient, and hν is the photon energy. The intersection with the x-axis of the plot of (αhν)^2^ versus hν corresponds to the optical gap E_g_. The bandgaps of CdS with precursor solution concentrations of 0.1 mol/L, 0.5 mol/L and 1.0 mol/L were determined to be 2.31 eV, 2.28 eV and 2.25 eV, respectively. These values are all smaller than the typical value for bulk CdS sample (about 2.4 eV) and this red shift increased with increasing starting-solution concentration. The red shift could originate from the surface defects of the CdS nanoparticles [31], that is because the defect states could underestimate bandgap energy for samples containing such defects.

**Figure 3:**
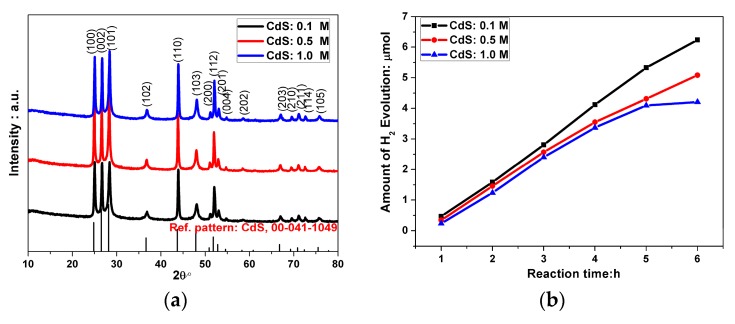
The crystal structure and performance. (**a**) X-ray diffraction patterns and (**b**) hydrogen evolution rate of CdS particles prepared at different starting-solution concentrations: 0.1 mol/L, 0.5 mol/L and 1.0 mol/L.

Figure 3a shows XRD patterns of the CdS particles obtained from different solution concentrations at a constant temperature. No effect of precursor concentration on the structural phase was found. The hexagonal phase of CdS particles with good crystallinity were obtained for all precursor concentrations. The capabilities of H_2_ evolution of CdS with different precursor concentrations are shown in Figure 3b. The results indicate that the activity of H_2_ evolution and the stability are clearly improved with the decrease of the CdS particle size, which could be due to a higher surface area in smaller CdS particles synthesized by a lower concentration precursor than that of larger ones.

### 2.2. Morphological, Optical and Structural Properties of Cu Doped CdS Particles

To investigate the doping effect on the photocatalytic activity of CdS particles, we doped CdS with Cu at doping levels of 0.2 mol %, 0.5 mol % and 1.0 mol %. to adjust its structural, electrical, and optical properties. In order to facilitate the identification of Cd, Cu and S in the Cu-doped CdS samples, X-ray fluorescence-spectrometry of 1.0% Cu-doped CdS was studied. To avoid the misleading that may result from the detection limit of the method, only results for 1.0% Cu-doped sample was presented. Table 1 shows XRF data of 1.0% Cu-doped CdS particles. From Table 1, one can see that the ratio of Cu to Cd is 0.01 which means that it is very precise to control element doping level by precursor composite adjustment in ultrasonic spray pyrolysis method. We can also find that there should be a high density of Cd vacancy (V_Cd_) as the mol ration of S is much higher than that of Cd. Since the formation energies of Cu substitutional defect (Cu_Cd_) is smaller than Cd vacancy (V_Cd_), presence of Cu in the sample is expected to eliminate the V_Cd_ defect [32].

**Figure 4:**
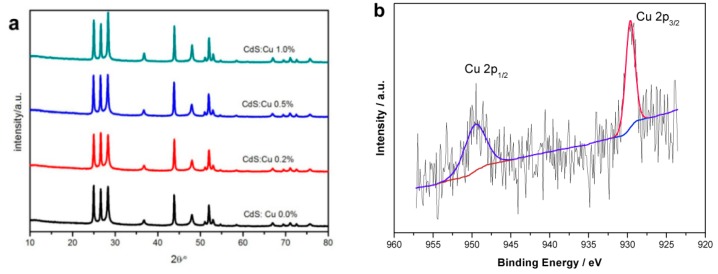
The crystal structure of doped CdS particles and electronic structure of dopant. (**a**) X-ray diffraction patterns of CdS particles of different Cu-dopants levels in the same concentration of starting-solution: 0.0%, 0.2%, 0.5% and 1.0%; (**b**) XPS profile of Cu^2+^ in 0.5% Cu-doped CdS particles.

**Figure 5:**
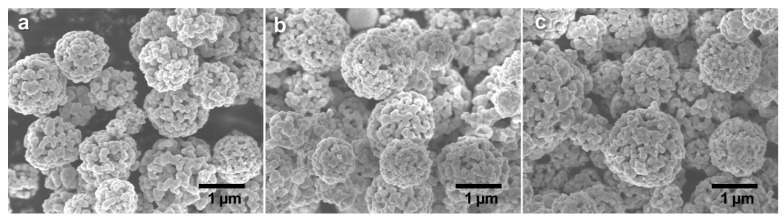
Scanning electron microscope images of Cu-doped prepared at various molar ratio of Cu/Cd: (**a**) 0.2%; (**b**) 0.5% and (**c**) 1.0%.

Figure 4a shows XRD patterns of CdS paticles of different Cu-dopants levels with ratio of Cu to Cd: 0.0%, 0.2%, 0.5% and 1.0%. No change of XRD pattern was observed between CdS particles with different Cu-doping levels, This could be due to the small doping level that does not affect the hexagonal phase of the CdS particles. The average crystalline sizes of Cu doped samples were estimated from the XRD patterns using the Scherrer equation [33]. The crystallite sizes calculated using the Scherrer equations are 111.5 nm, 77.4 nm, 77.0 nm and 76.8 nm for 0%, 0.2%, 0.5% and 1% doped samples, respectively. The decrease of crystallite size could result from the presence of Cu in the doped samples which causes restriction to the growth of CdS nanoparticles during the pyrolysis process [34]. To further investigate valence state of Cu, XPS is carried out and the XPS profile of Cu 2p is presented in Figure 4b. The Cu 2p_1/2_ (949.6 eV) and Cu 2p_3/2_ (929.6 eV) lines verify that the Cu exists as Cu^2+^ [35]. The SEM images of CdS particles of different Cu-dopants levels were also obtained to check the doping influence on the morphology and size of CdS particles. It was clearly shown in Figure 5 that the doping increased the porosity compared to the undoped one as shown in Figure 1b. However, the porosity showed no significant changes at different doping level and the average particle sizes scarcely changed for different samples.

**Figure 6:**
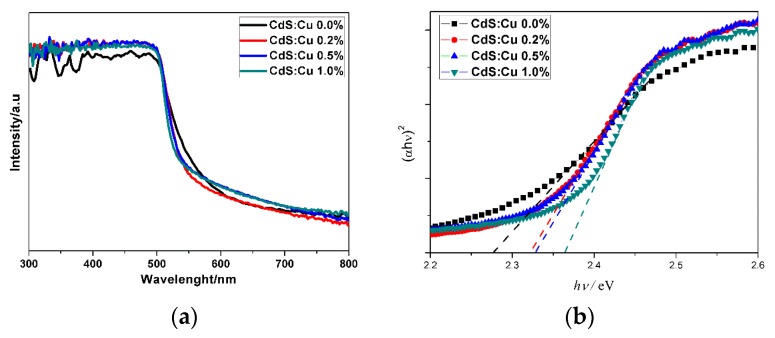
The optical properties of doped CdS particles. (**a**) UV-Vis absorption spectra and; (**b**) Tauc plot of Cu-doped prepared at various molar ratio of Cu/Cd: 0.0%, 0.2%, 0.5% and 1.0%.

The influence of Cu doping on the band gaps of CdS particles were investigated by the UV-Vis absorption spectra. Figure 6 shows the UV-Vis absorption spectra of Cu-doped prepared by ultrasonic spray pyrolysis at various molar ratio of Cu/Cd. It can be seen that all the samples show a visible-light absorption region from 500 nm to 560 nm, and slight blue shifts of the absorption edge of Cu-doped CdS particles were observed comparing to pure CdS particles. The band gaps of pure CdS particles and Cu-doped CdS can be calculated by the Tauc plots as shown in Figure 6b. It was found that the band gaps of doped CdS increased with higher Cu doping concentration. Since copper sulfide possesses a lower band gap compared to CdS, any possibility of CuS formation can be ruled out. The Cu element did not decrease the band gaps of CdS by forming new levels; however, it can cause the band gap increase which could originate from quantum effects of the decreasing of crystallite size after Cu doping [36,37]. The high density defect of CdS nanoparticles synthesized by pyrolysis method narrowed the band gaps of CdS (shown in Figure 2) by creating defect states in the band gap, while Cu doping increased the band gaps of CdS particles which shift the band gaps back towards the typical value for bulk CdS sample.

**Figure 7:**
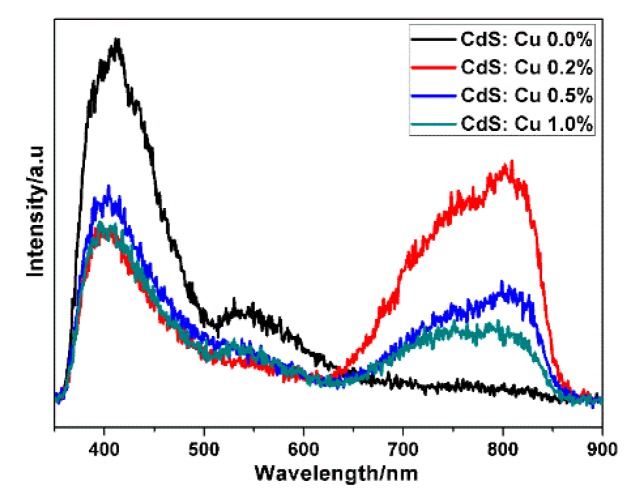
Photoluminescence spectra of the different levels Cu-doped CdS particle: 0.0%, 0.2%, 0.5% and 1.0% (excitation wavelength: 340 nm).

Photoluminescence (PL) spectra were used to study the optical characteristic of CdS after doping. PL spectra of the CdS particle with different doping levels were recorded with a 340 nm excitation wavelength and the results are shown in Figure 7. For pure CdS, it is obvious that there is a PL peak centered at 410 nm, this peak is the excitonic emission of CdS [34]. Another peak for pure CdS is a peak centered at 550 nm in the section of visible light. This peak can be attributed to charge carrier recombination at surface states of obtained CdS [34]. This recombination was reported as a radiative recombination of free charge carrier and trapped charge carriers at surface defects [38,39].

The surface states in CdS could arise from sulfur vacancies or cadmium vacancies. According to the rich sulfur source synthesis condition and XRF results, the surface states could result from Cd vacancies, which can act as hole trap states [40]. For the Cu-doped CdS particles, excitonic emissions as well as surface state emission decreased after Cu doping; however, a new broad peak range from 650 nm to 850 nm emerged. This emission peak is clearly a Cu-related emission, which could be the Cu acceptor level (T2 level) originated from the triplet state of Cu^2+^ [41]. With increase of doping level from 0.2% to 1%, this emission intensity decreased which could be the result of increasing nonradiative transitions from T2 level to the valence band of CdS with increase of Cu concentration in CdS [34].

**Figure 8:**
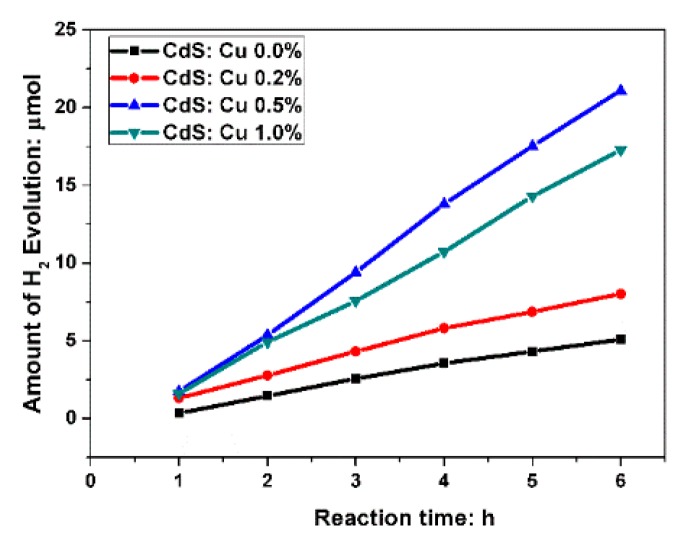
Amount of hydrogen evolution form water with different Cu-doping levels CdS particles: 0.0%, 0.2%, 0.5% and 1.0%.

The amount of hydrogen evolution with different Cu-doping levels of CdS particles is shown in Figure 8. It can be seen that hydrogen production rate of all three Cu-doped CdS samples are higher than that of pure CdS, and the 0.5% Cu-doped sample shows the highest hydrogen production rate. Doping is often used to improve absorption efficiency by shifting absorption edge to visible light. However, in our case, Cu doping did not narrow the bandgaps of CdS, as discussed in the previous section. It was reported that the Cd^2+^ are substituted by Cu^+^ in unit cells of CdS [42]. With the increase of Cu doping, there will be an increase of Cu atoms substituted for Cd sites, acting as acceptors and giving rise to p type conductivity [43]. At the same time, surfaces with a state originating from Cd vacancy and acting as recombination center decreased, and thus the photocurrent improved. When the doping level is higher than 0.5 mol %, a further increase of Cu doping increased the nonradiative transitions from T2 level to the valence band of CdS and caused a higher charge recombination rate at the Cu sites and thus a decrease of photocatalytic performance.

## 3. Materials and Methods

**Figure 9:**
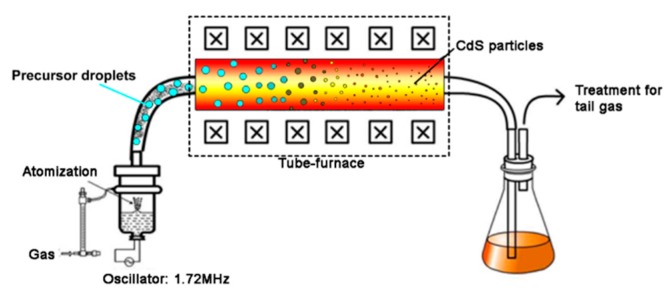
Schematic drawing of ultrasonic spray pyrolysis system for synthesis of Cu doped CdS.

Analytical grade Cd(NO_3_)_2_·4H_2_O, SC(NH_2_)_2_ and Cu(NO_3_)_2_·3H_2_O were used as received. The precursor for each spray pyrolysis synthesis of undoped CdS particle was prepared by dissolving Cd(NO_3_)_2_·4H_2_O and SC(NH_2_)_2_ in 100 mL deionized water to reach a concentration of 0.5 mol/L (pH = 4.1) in a beaker. For the precursor for spray pyrolysis synthesis of metal doped CdS particle, certain amount of metal (Cu, Ni or Pb) nitrates were added into obtained 100 mL precursors to make the metal (Cu, Ni or Pb) to Cd molar ratio at 0.005. The CdS particles with different Cu doping level (Cu to Cd molar ratio: 0.002, 0.005 and 0.01) were also synthesized by adding different amount of Copper nitrate. The obtained precursors for different samples were transferred to a custom-made ultrasonic nebulizer (with a frequency of 1.72 MHz), in which the precursor was atomized into micro-droplet and carried into a 500 °C tube-furnace by flowing Nitrogen. Upon pyrolysis reaction, CdS or doped CdS spherical micro-particles were formed and settled on the inner wall of the tube-furnace. The micro-particles were then collected, washed with ethanol and dried in vacuum at 100 °C for about 8 h. The schematic diagram of ultrasonic spray pyrolysis system for CdS spherical micro-particles preparation is shown in Figure 9.

The structural and optical properties of CdS particles obtained by ultrasonic spray pyrolysis method were examined by scanning electron microscopy (SEM, model JSM-6700, JEOL, Tokyo, Japan), X-ray fluorescence-spectrometry (XRF, 4KW, RhKα Bruker AXS, Karlsruhe, Germany), UV-Visible spectrophotometer (Hitachi U-4100, Tokyo, Japan), X-ray diffraction (CuKα, 40 KV, 40 mA, PANalytical BV, Almelo, The Netherlands) and Photoluminescence (PTI QuantaMaster^TM^ 40, Photon Technology International, Lawrenceville, NJ, USA).

Photocatalytic hydrogen evolution was performed in a gas-closed system with a side irradiation Pyrex cell at 35 °C. The 12.56 cm^2^ side window of the cell was irradiated with a PLS-SXE300/300UV Xe lamp through a cutoff filter (>430 nm, T = 65%). 0.2 g photocatalysts powder was dispersed by a magnetic stirrer in an aqueous solution (200 mL) with a pH of 13.2 containing Na_2_SO_3_ (0.25 mol/L) and Na_2_S (0.35 mol/L) as electron donors in the cell. The amount of H_2_ evolved was determined by sampling from the cell and test in a thermal conductivity detector (TCD) gas chromatography (Beifen-Ruili SP-2100, NaX zeolite column, nitrogen as a carrier gas). Blank experiments revealed that no hydrogen was produced without the catalyst being added or without light irradiation. The principle for evaluation of photocatalytic activity followed the reported description of Zhang [15].

## 4. Conclusions

Ultrasonic spray pyrolysis was successfully applied for the preparation of spherical particles. CdS spherical particles with different starting-solution concentrations and Cu doped CdS with doping levels of 0.2%, 0.5% and 1.0% have been considered to study the properties of photocatalytic hydrogen production. The optical absorption to UV-visible light, PL spectrum, morphology characteristics, XRF, XRD analysis and the hydrogen production rates were investigated. The photocatalytic activity of CdS has been improved by Cu doping and the optimal doping level was found to be 0.5 mol % in the investigated range. In addition, a decreased band gap of CdS synthesized with this method was found and compared to typical value for bulk CdS sample. We ascribed the decrease of bandgap to defect levels formed in the band gap that originated from Cd vacancies. The enhancement of photocatalytic performance by Cu doping could result from the reducing of defect states by incorporating Cu elements, which take the positions of Cd vacancies. The spray pyrolysis is a convenient and effective method for preparing the doped CdS particles, which provide a solution-based technology platform to prepare large scale semiconductor composition or nanostructures for efficient photocatalytic applications.

## Figures and Tables

**Table 1:** X-ray fluorescence-spectrometry (XRF) analysis result of 1.0% Cu-doped CdS.

Title	Cu	Cd	S	O
Intensity (KCps)	11.0	346.3	223.3	0.1
wt %	0.385	66.7	27.5	5.46
mol %	0.335	32.965	47.74	18.957

## References

[1] Fujishima A., Honda K. (1972). Electrochemical Photolysis of Water at a semiconductor Electrode. Nature.

[2] Zhang K., Guo L. (2013). Metal sulphide semiconductors for photocatalytic hydrogen production. Catal. Sci. Technol..

[3] Chen Y., Feng X., Liu M., Su J., Shen S. (2016). Towards efficient solar-to-hydrogen conversion: Fundamentals and recent progress in copper-based chalcogenide photocathodes. Nanophotonics.

[4] Chen J., Wu X., Yin L., Li B., Hong X., Fan Z., Chen B., Xue C., Zhang H. (2015). One-pot Synthesis of CdS Nanocrystals Hybridized with Single-Layer Transition-Metal Dichalcogenide Nanosheets for Efficient Photocatalytic Hydrogen Evolution. Angew. Chem. Int. Ed..

[5] Vaquero F., Fierro J.L.G., Navarro Yerga R.M. (2016). From Nanorods to Nanowires of CdS Synthesized by a Solvothermal Method: Influence of the Morphology on the Photoactivity for Hydrogen Evolution from Water. Molecules.

[6] Sakimoto K.K., Wong A.B., Yang P. (2016). Self-photosensitization of nonphotosynthetic bacteria for solar-to-chemical production. Science.

[7] Yu J., Yu Y., Zhou P., Xiao W., Cheng B. (2014). Morphology-dependent photocatalytic H_2_-production activity of CdS. Appl. Catal. B Environ..

[8] Xiang Q., Cheng B., Yu J. (2013). Hierarchical porous CdS nanosheet-assembled flowers with enhanced visible-light photocatalytic H_2_-production performance. Appl. Catal. B Environ..

[9] Jin J., Yu J., Liu G., Wong P.K. (2013). Single crystal CdS nanowires with high visible-light photocatalytic H_2_-production performance. J. Mater. Chem. A.

[10] Zhang N., Yang M., Tang Z., Xu Y. (2013). CdS-graphene nanocomposites as visible light photocatalyst for redox reactions in water: A green route for selective transformation and environmental remediation. J. Catal..

[11] Long L., Yu X., Wu L., Li J., Li X. (2014). Nano-CdS confined within titanate nanotubes for efficient photocatalytic hydrogen production under visible light illumination. Nanotechnology.

[12] Fu J., Chang B., Tian Y., Xi F., Dong X. (2013). Novel C_3_N_4_-CdS composite photocatalysts with organic-inorganic heterojunctions: *In situ* synthesis, exceptional activity, high stability and photocatalytic mechanism. J. Mater. Chem. A.

[13] Moriya M., Minegishi T., Kumagai H., Katayama M., Kubota J., Domen K. (2013). Stable Hydrogen Evolution from CdS-Modified CuGaSe_2_ Photoelectrode under Visible-Light Irradiation. J. Am. Chem. Soc..

[14] Lingampalli S.R., Gautam U.K., Rao C.N.R. (2013). Highly efficient photocatalytic hydrogen generation by solution-processed ZnO/Pt/CdS, ZnO/Pt/Cd_1−x_Zn_x_S and ZnO/Pt/CdS_1_−_x_Se_x_ hybrid nanostructures. Energy Environ. Sci..

[15] Zhang K., Jing D., Xing C., Guo L. (2007). Significantly improved photocatalytic hydrogen production activity over Cd_1−x_Zn_x_S photocatalysts prepared by a novel thermal sulfuration method. Int. J. Hydrog. Energy.

[16] Liu M., Jing D., Zhou Z., Guo L. (2013). Twin-induced one-dimensional homojunctions yield high quantum efficiency for solar hydrogen generation. Nat. Commun..

[17] Bao N., Shen L., Takata T., Domen K. (2008). Self-Templanted Synthesis of Nanoporous CdS Nanostructures for Highly Efficienct Photocatalytic Hydrogen Production under Visible Light. Chem. Mater..

[18] Zang J., Zhao G., Han G. (2007). Preparation of CdS Nanopaticles by Hydrothemal Method in Microemulsion. Front. Chem. China.

[19] Murali G., Reddy D.A., Sambasivam S., Vijayalakshmi R.P., Reddy V.R. (2014). CdS microflowers and interpenetrated nanorods grown on Si substrate: Structural, optical properties and growth mechanism. Mater. Chem. Phys..

[20] Tripathi R.M., Bhadwal A.S., Singh P., Shrivastav A., Singh M.P., Shrivastav B.R. (2014). Mechanistic aspects of biogenic synthesisof CdS nanoparticles using *Bacillus licheniformis*. Adv. Nat. Sci. Nanosci. Nanotechnol..

[21] Zhou L., Hu X., Wu S. (2013). Effects of deposition temperature on the performance of CdS films with chemical bath deposition. Surf. Coat. Tech..

[22] Okuyama K., Lenggoro I.W., Tagami N., Tamaki S., Tohge N. (1997). Preparation of ZnS and CdS fine particles with different particle sizes by a spray-pyrolysis method. J. Mater. Sci..

[23] Huang J., Cheuk W., Wu Y., Lee F.S., Ho W. (2012). Template-free synthesis of ternary sulfides submicrospheres as visible light photocatalysts by ultrasonic spray pyrolysis. Catal. Sci. Technol..

[24] Sunil M.A., Deepa K.G., Nagaraju J. (2014). Growth of AgInS_2_ thin films by ultrasonic spray pyrolysis technique. Thin Solid Films.

[25] Korake P.V., Achary S.N., Gupta N.M. (2015). Role of aliovalent cation doping in the activity of nanocrystalline CdS for visible-light-driven H_2_ production from water. Int. J. Hydrog. Energy.

[26] Chen X., Burda C. (2008). The electronic origin of the visible-light absorption properties of C-, *N*- and *S*-doped TiO_2_ nanomaterials. J. Am. Chem. Soc..

[27] Huang S., Lin Y., Yang J., Li X., Zhang J., Yu J., Shi H., Wang W., Yu Y. (2013). Enhanced photocatalytic activity and stability of semiconductor by Ag doping and simultaneous deposition: The case of CdS. RSC Adv..

[28] Xie R., Su J., Li M., Guo L. (2013). Structural and Photoelectrochemical Properties of Cu-Doped CdS Thin Films Prepared by Ultrasonic Spray Pyrolysis. Int. J. Photoenergy.

[29] Rmili A., Ouachtari F., Bouaoud A., Louardi A., Chtouki T., Elidrissi B., Erguig H. (2013). Structural, optical and electrical properties of Ni-doped CdS thin films prepared by spray pyrolysis. J. Alloy. Compd..

[30] Tauc J. (1974). Amorphous And Liquid Semiconductors.

[31] Cortes A., Gomez H., Marotti R.E., Riveros G., Dalchiele E.A. (2004). Grain size dependence of the bandgap in chemical bath deposited CdS thin films. Sol. Energy Mater. Sol. Cells.

[32] Wei S., Zhang S., Zunger A. (2000). First-principles calculation of band offsets, optical bowings, and defects in CdS, CdSe, CdTe, and their alloys. J. Appl. Phys..

[33] Scherrer P. (1918). Estimation of the size and internal structure of colloidal particles by means of röntgen. Nachr. Ges. Wiss. Göttingen.

[34] Mandal P., Talwar S.S., Major S.S., Srinivasa R.S. (2008). Orange-red luminescence from Cu doped CdS nanophosphor prepared using mixed Langmuir-Blodgett multilayers. J. Chem. Phys..

[35] Wang L., Huang S., Sun Y. (2013). Low-temperature synthesis of hexagonal transition metal ion doped ZnS nanoparticles by a simple colloidal method. Appl. Surf. Sci..

[36] Nakanishi T., Ohtani B., Uosaki K. (1998). Fabrication and char-acterization of CdS-nanoparticle mono- and multilayers on a self-assembled monolayer of alkanedithiols on gold. J. Phys. Chem. B.

[37] Wang Y., Suna A., Mahler W., Kosowski R. (1987). PbS in polymers. From molecules to bulk solids. J. Chem. Phys..

[38] Chen H., Huang X., Xu L., Xu J., Chen K., Feng D. (2000). Self-assembly and photoluminescence of CdS-mercaphtoacetic clusters with internal structures. Superlattice Microst..

[39] Chen W., Xu Y., Lin Z., Wang Z., Lin L. (1998). Formation, structure and fluorescence of CdS clusters in a mesoporous zeolite. Solid State Commun..

[40] Costa V.C., Shen Y., Bray K.L. (2002). Luminescence properties of nanocrystalline CdS and CdS: Mn^2+^ doped silica-type glasses. J. Non-Cryst. Solids.

[41] Panda R., Rathore V., Rathore M., Shelke V., Badera N., Sharath Chandra L.S., Jain D., Gangrade M., Shripati T., Ganesan V. (2012). Carrier recombination in Cu doped CdS thin films: Photocurrent and optical studies. Appl. Surf. Sci..

[42] Abe T., Kashiwaba Y., Baba M., Imai J., Sasaki H. (2001). XPS analysis of p-type Cu-doped CdS thin films. Appl. Surf. Sci..

[43] Kashiwaba Y., Kanno I., Ikeda T. (1992). p-Type characteristics of Cu-doped CdS thin films. Jpn. J. Appl. Phys..

